# Supplementary material for: Stage-Dependent Increase of Systemic Immune Activation and CCR5+CD4+ T Cells in Filarial Driven Lymphedema in Ghana and Tanzania
Source: Pathogens. 2023 Jun 7;12(6):809. doi: 10.3390/pathogens12060809 (PMC10301430; doi:10.3390/pathogens12060809)
Supplement: Supplementary file 1 [file pathogens-12-00809-s001.zip › Supplementary Figures S1 and S2.pptx]

## Slide 1
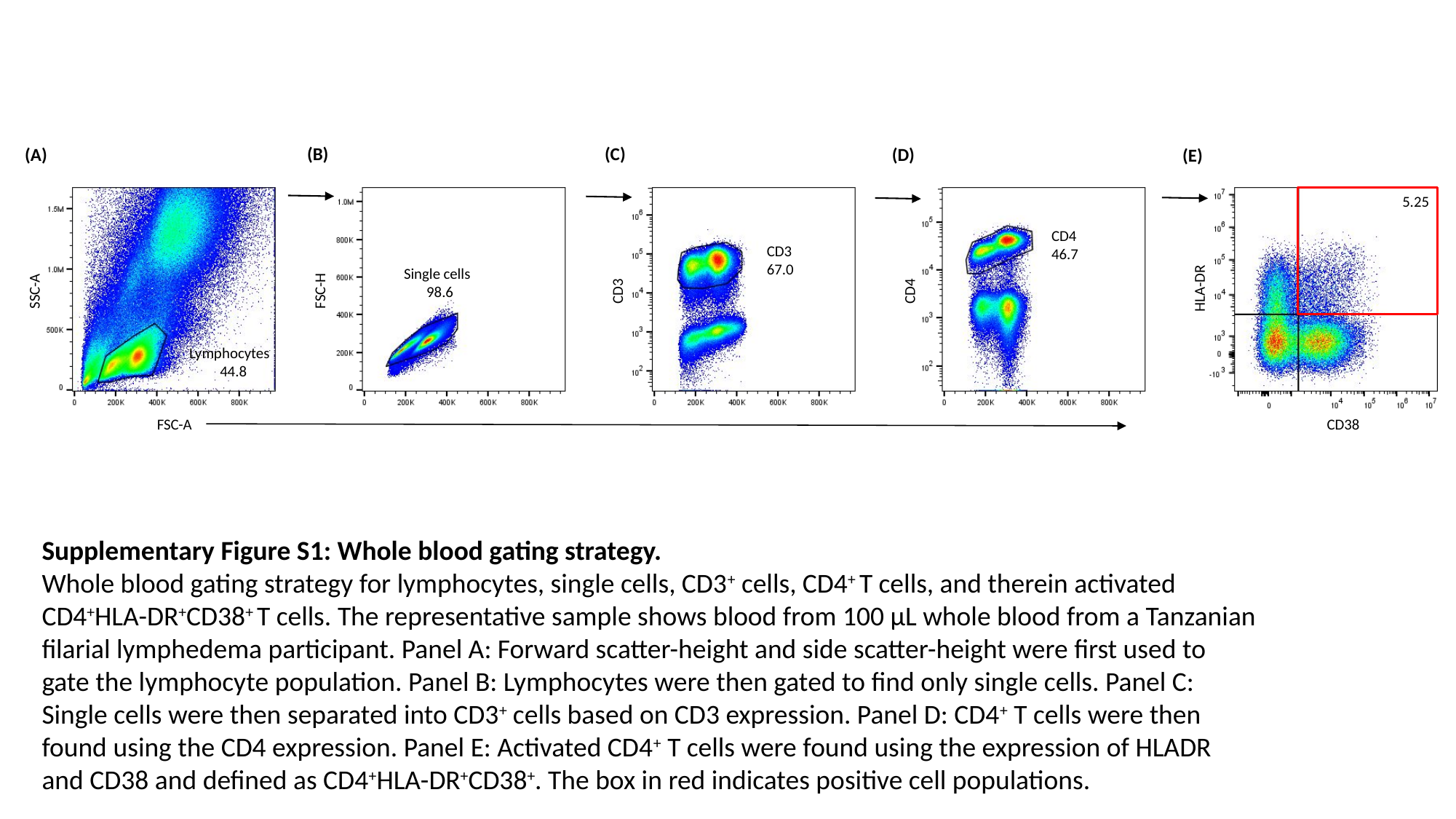

(C)
(B)
(D)
(A)
(E)
5.25
CD4
46.7
CD3
67.0
Single cells
98.6
CD4
CD3
FSC-H
SSC-A
HLA-DR
Lymphocytes
44.8
FSC-A
CD38
Supplementary Figure S1: Whole blood gating strategy.
Whole blood gating strategy for lymphocytes, single cells, CD3+ cells, CD4+ T cells, and therein activated CD4+HLA-DR+CD38+ T cells. The representative sample shows blood from 100 µL whole blood from a Tanzanian filarial lymphedema participant. Panel A: Forward scatter-height and side scatter-height were first used to gate the lymphocyte population. Panel B: Lymphocytes were then gated to find only single cells. Panel C: Single cells were then separated into CD3+ cells based on CD3 expression. Panel D: CD4+ T cells were then found using the CD4 expression. Panel E: Activated CD4+ T cells were found using the expression of HLADR and CD38 and defined as CD4+HLA-DR+CD38+. The box in red indicates positive cell populations.

## Slide 2
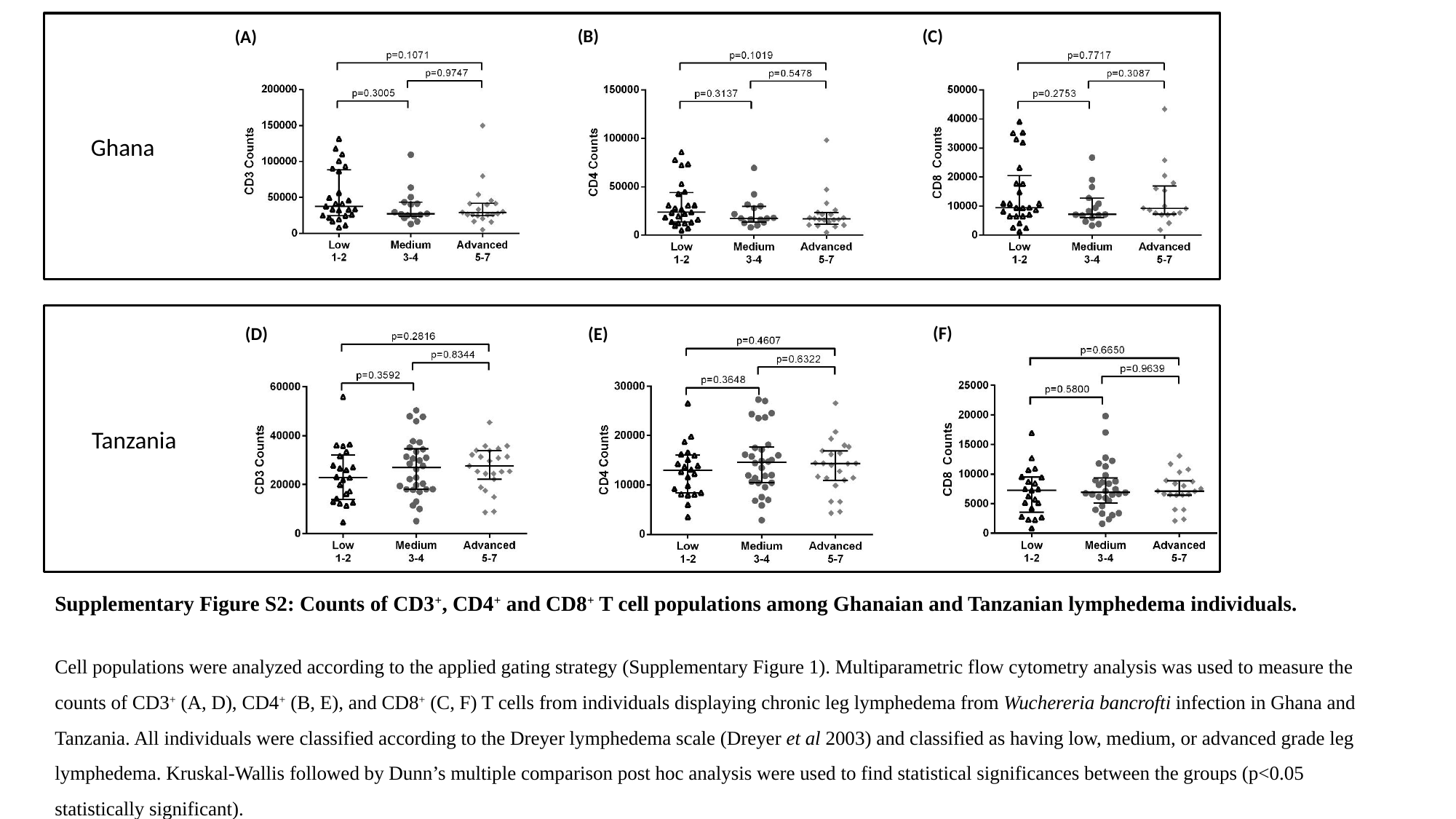

(C)
(B)
(A)
Ghana
(F)
(E)
(D)
Tanzania
Supplementary Figure S2: Counts of CD3+, CD4+ and CD8+ T cell populations among Ghanaian and Tanzanian lymphedema individuals.
Cell populations were analyzed according to the applied gating strategy (Supplementary Figure 1). Multiparametric flow cytometry analysis was used to measure the counts of CD3+ (A, D), CD4+ (B, E), and CD8+ (C, F) T cells from individuals displaying chronic leg lymphedema from Wuchereria bancrofti infection in Ghana and Tanzania. All individuals were classified according to the Dreyer lymphedema scale (Dreyer et al 2003) and classified as having low, medium, or advanced grade leg lymphedema. Kruskal-Wallis followed by Dunn’s multiple comparison post hoc analysis were used to find statistical significances between the groups (p<0.05 statistically significant).
